# *Anguillicola crassus* Infection Significantly Affects the Silvering Related Modifications in Steady State mRNA Levels in Gas Gland Tissue of the European Eel

**DOI:** 10.3389/fphys.2016.00175

**Published:** 2016-05-23

**Authors:** Bernd Pelster, Gabriel Schneebauer, Ron P. Dirks

**Affiliations:** ^1^Institute of Zoology, University of InnsbruckInnsbruck, Austria; ^2^Center for Molecular Biosciences, University of InnsbruckInnsbruck, Austria; ^3^ZF-ScreensLeiden, Netherlands

**Keywords:** swimbladder function, spawning migration, nematode infections, European eel, silvering

## Abstract

Using Illumina sequencing, transcriptional changes occurring during silvering in swimbladder tissue of the European eel have been analyzed by comparison of yellow and silver eel tissue samples. Functional annotation analysis based on GO terms revealed significant expression changes in a number of genes related to the extracellular matrix, important for the control of gas permeability of the swimbladder, and to reactive oxygen species (ROS) defense, important to cope with ROS generated under hyperbaric oxygen partial pressures. Focusing on swimbladder tissue metabolism, levels of several mRNA species encoding glucose transport proteins were several-fold higher in silver eels, while enzymes of the glycolytic pathway were not affected. The significantly higher steady state level of a transcript encoding for membrane bound carbonic anhydrase, however, suggested that CO_2_ production in the pentose phosphate shunt and diffusion of CO_2_ was of particular importance in silver eel swimbladder. In addition, the mRNA level of a large number of genes related to immune response and to sexual maturation was significantly modified in the silver eel swimbladder. The modification of several processes related to protein metabolism and transport, cell cycle, and apoptosis suggested that these changes in swimbladder metabolism and permeability were achieved by increasing cell turn-over. The impact of an infection of the swimbladder with the nematode *Anguillicola crassus* has been assessed by comparing these expression changes with expression changes observed between uninfected yellow eel swimbladder tissue and infected silver eel swimbladder tissue. In contrast to uninfected silver eel swimbladder tissue, in infected tissue the mRNA level of several glycolytic enzymes was significantly elevated, and with respect to extracellular matrix, several mucin genes were many-fold higher in their mRNA level. Modification of many immune related genes and of the functional categories “response to DNA damage stimulus” and “cellular response to stress” illustrated the damaging effect of the nematode infection. This study has identified a range of cellular processes in the swimbladder of silver eels that appear to be altered by nematode infection. These altered cellular processes could contribute to detrimental changes in swimbladder function that, in turn, may lead to impairment of spawning migration.

## Introduction

The European eel *Anguilla anguilla* is a catadromous fish spending most of its lifetime as yellow eel in the European freshwater system, and returning to the Sargasso Sea for reproduction. The spawning migration is a journey of about 5000–7000 km from the European coast to the Sargasso Sea, taking about 3.5–6 months. Eels do not feed during this journey and on-board fuels must be sufficient to support the journey. Recent studies revealed that migrating eels perform daily vertical migrations swimming at a depth of about 100–300 m at night time, but at a depth of 600–1000 m at day time (Aarestrup et al., 2009; Wysujack et al., 2015). The concomitant changes in hydrostatic pressure directly affect pressure and volume of the swimbladder, which is used as a buoyancy organ.

Anguillidae are physostomatous fish with a persisting ductus pneumaticus, but in the European eel the ductus pneumaticus is functionally closed, and converted to a resorbing bladder (Dorn, 1961; Pelster, 2013). Accordingly, eels cannot gulp air and the swimbladder is filled by diffusion of gas, mainly oxygen and CO_2_, from the blood into the swimbladder lumen. The increase in oxygen and CO_2_ partial pressures required to drive the diffusion of these gas molecules into the swimbladder is achieved by acidification of the blood via lactic acid and CO_2_ release from swimbladder gas gland cells, which reduces the oxygen carrying capacity of the hemoglobin. This so-called single concentrating effect is subsequently multiplied by countercurrent multiplication in the rete mirabile (Pelster and Randall, 1998; Pelster, 2009, 2013).

Compared with the hydrostatic pressure changes eels encounter during the diurnal vertical migrations in the ocean (21 atm at a depth of 200 m and 101 atm at a depth of 1000 m), the pressure changes eels encounter in the European freshwater system are probably only small. It therefore is not surprising that the process of silvering, which prepares the freshwater adapted yellow eel for the spawning migration in the ocean, also affects the swimbladder. During silvering the *retia mirabilia* are significantly enlarged, indicating an improvement of the countercurrent concentrating ability. In addition, swimbladder wall thickness and the swimbladder vascularization increase. Guanine deposition in the eel swimbladder wall is enhanced, which decreases its gas permeability and thus reduces diffusional gas loss (Kleckner, 1980a,b; Yamada et al., 2001). In the American eel *Anguilla rostrata*, a five-fold increase in the rate of gas deposition has been recorded in silver eels (Kleckner, 1980a). It therefore is assumed that these silvering induced changes are connected to a significant improvement in swimbladder function (Sebert et al., 2009; Righton et al., 2012).

While the metabolic processes initiating gas secretion have been repeatedly addressed (Steen, 1963; Kobayashi et al., 1990; Pelster, 1995b, 2013), the molecular changes underlying and controlling these silvering events in gas gland cells are completely unknown. Furthermore, the influence of an infection of the swimbladder with the nematode *Anguillicola crassus*, which has spread over Europe in the last few decades and infected most of the European eels (Lefebvre et al., 2013), on gene expression in gas gland cells has not yet been addressed. It has been suspected, however, that the infection may influence the silvering process (Fazio et al., 2012).

In this study, we therefore hypothesized that silvering was connected to large changes in the transcription of genes related to swimbladder function. We particularly focused on transcriptional changes of genes related to (1) glucose metabolism and (2) ion exchange, which are required for acid production and release in order to switch on the Root effect for gas secretion (Pelster and Randall, 1998; Pelster, 2013); (3) angiogenesis, required for appropriate blood supply to the swimbladder (Kleckner, 1980a); (4) ROS defense, required to avoid oxidative stress related to hyperbaric oxygen tensions (Morris and Albright, 1981, 1984; Pelster, 2001; Lushchak and Semchyshyn, 2012); (5) extracellular matrix, involved in reducing diffusional gas loss from the swimbladder (Kleckner, 1980a,b; Yamada et al., 2001); (6) immune response, required to defeat the nematode infection (Lefebvre et al., 2011, 2013); and (7) maturation, which occurs in silver eels during spawning migration (Dufour et al., 2003). As a second step, we hypothesized that these transcriptional changes would be modified by an infection of the swimbladder with the nematode *A. crassus*.

## Materials and methods

Experiments were performed with European eels *A. anguilla*. Yellow eels were collected by local fishermen in Lake Constance with bottom traps. They were kept in the freshwater aquarium of the Institute of Zoology at the University of Innsbruck until sampling the tissue. Eels with no or one small *A. crassus* in the swimbladder without visible modification of the swimbladder wall were accepted as uninfected eels (*N* = 5, body mass 515.0 ± 146.4 g). Silver eels were collected by local fishermen in the IJsselmeer and kept in large tanks at Leiden University until sampling the tissue. Recent studies have shown that the European eel is panmictic (Als et al., 2011; Jacobsen et al., 2014), therefore the results of our study should not be biased by the different sampling points. Table 1 summarizes the morphometrics of uninfected and infected silver eels. Body mass of uninfected silver eels was 1437.2 ± 1182.7 g (*N* = 5), and body mass of infected eels was 830.8 ± 150.8 g (*N* = 6). In infected swimbladders between 5 and 30 full grown parasites were detected, and the swimbladder wall was thickened and opaque, as described previously (Würtz and Taraschewski, 2000). Completing one infection cycle requires 3–4 month, suggesting that theses eels have been infected with *A. crassus* for a long time (Kirk, 2003). The silver index calculated according to Durif et al. (2005) was similar in both groups with 4.0 ± 0.8 for uninfected silver eels and 4.1 ± 0.8 for infected silver eels. Eels were killed with an overdose of neutralized MS222. The swimbladder was dissected and the swimbladder epithelium (consisting of gas gland cells) was carefully freed from connective tissue. The epithelium was shock frozen in liquid nitrogen and stored at −80°C until further use. Tissue sampling was performed in compliance with the Austrian law, the guidelines of the Austrian Federal Minister for Education, Arts and Culture, and also the Dutch law, and were approved by the animal experimental committee of Leiden University.

**Table 1:** **Morphometrics of uninfected and infected silver eels including silver index, calculated according to Durif et al. (2005)**.

	**Silver uninfected**	**Silver infected**
Body mass (g)	1437 ± 1182	830 ± 151
Body length (cm)	82.7 ± 15.2	73.2 ± 5.9
Pectoral fin length (mm)	38.1 ± 5.7	36.1 ± 3.0
Horizontal eye diameter (mm)	10.4 ± 1.9	10.0 ± 0.9
Vertical eye diameter (mm)	10.4 ± 1.8	9.8 ± 0.4
Silver index	4.0 ± 0.8	4.1 ± 0.8

*There was no significant difference between uninfected (N = 5) and infected (N = 6) silver eels*.

## RNA isolation and illumina RNAseq analysis

Total RNA was isolated from gas gland tissue using the Qiagen miRNeasy kit according to the manufacturer's instructions (Qiagen, Venlo, Netherlands). Quality and integrity of the RNA were checked on an Agilent Bioanalyzer 2100 total RNA Nano series II chip (Agilent, Amstelveen, Netherlands). Illumina RNAseq libraries were prepared from 2 μg total RNA using the Illumina TruSeq™ RNA Sample Prep Kit v2 according to the manufacturer's instructions (Illumina Inc., San Diego, CA, USA). All RNAseq libraries (150–750 bp inserts) were sequenced on an Illumina HiSeq2000 sequencer as 2 × 50 nucleotides paired-end reads according to the manufacturer's protocol. Image analysis and base calling were done using the Illumina pipeline.

### Illumina data processing

Data processing was performed as described previously (Dirks et al., 2014; Burgerhout et al., 2016). Briefly, reads (10–20 million per sample) were aligned to the draft genome sequence of European eel (Henkel et al., 2012) using TopHat (version 2.0.5; Trapnell et al., 2009). Secondary alignments of reads were excluded by filtering the files using SAMtools (version 0.1.18; Li et al., 2009). Aligned fragments per predicted gene were counted from SAM alignment files using the Python package HTSeq (version 0.5.3p9; Anders et al., 2015). In order to make comparisons across samples possible, these fragment counts were corrected for the total amount of sequencing performed for each sample. As a correction scaling factor, library size estimates determined using the R/Bioconductor (release 2.11) package DESeq (Anders and Huber, 2010) were employed. Read counts were normalized by dividing the raw counts obtained from HTSeq by its scale factor. Detailed read coverage for individual genes was extracted from the TopHat alignments using SAMtools.

Differences in the steady state mRNA level between yellow and silver eels and also between yellow and infected silver eels were identified using DESeq, the cut-off for significance was set to *P* < 0.01. GO enrichment analysis was performed using Database for Annotation, Visualization and Integrated Discovery (DAVID) software tools (version 6.7; https://david.ncifcrf.gov). An EASE score of 0.05 along with standard default settings was used, and the resulting categories were considered significant at *P* < 0.05. Gene ontology annotations were used for a detailed pathway and biological process analysis of mRNA transcripts with different steady state level.

## Results

### General observations

**Figure 1:**
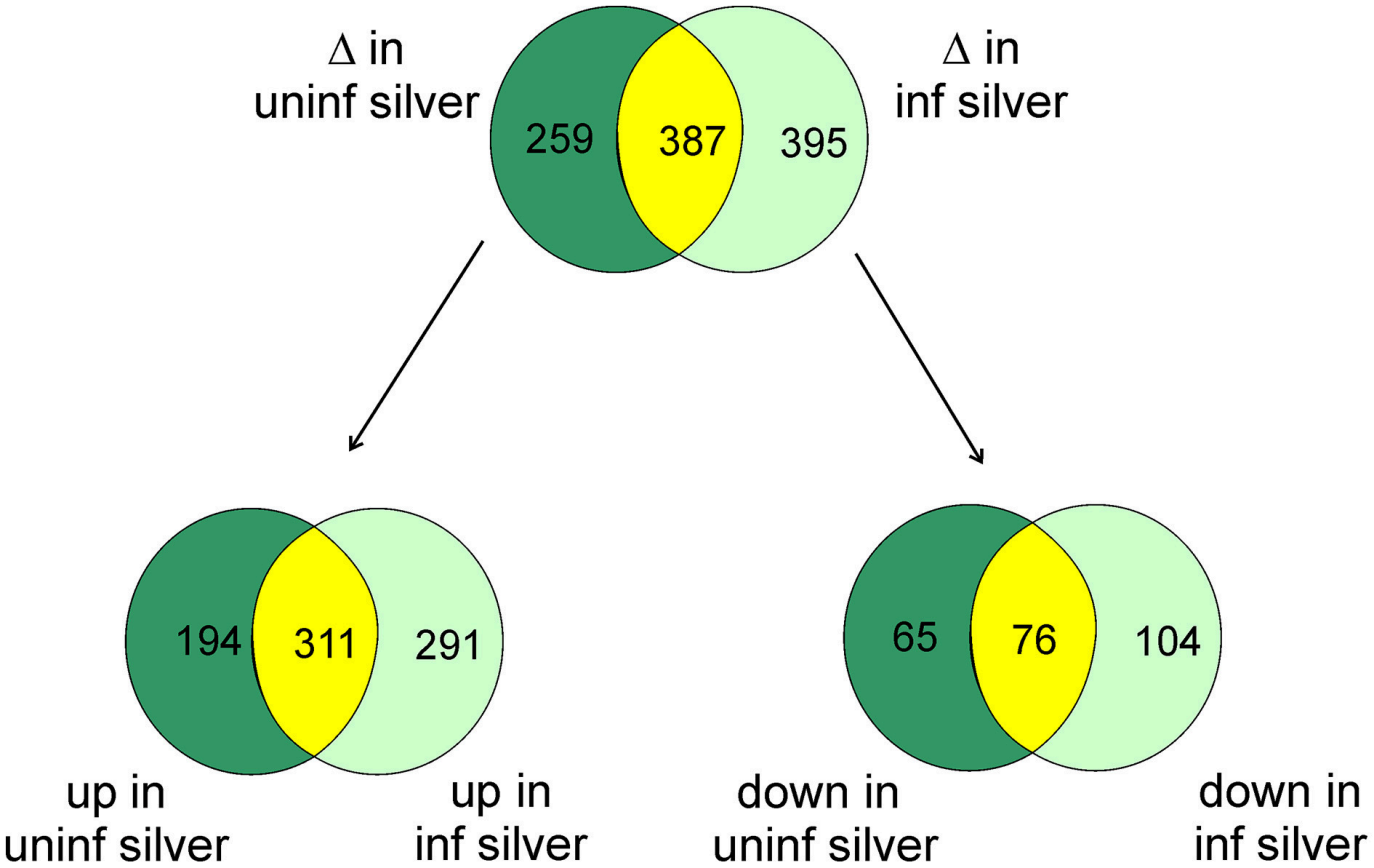
**Venn diagram showing the total number of differentially transcribed genes participating in defined pathways expected to be involved in swimbladder function and/or silvering in uninfected silver eels (dark green) and in infected silver eels (light green), and the number of genes affected in both groups (yellow) as compared with uninfected yellow eels**. The lower part shows the number of genes either up- or down-regulated.

At the significance level of *P* < 0.01 in uninfected silver eels 646 genes were significantly different in their transcription level as compared with uninfected yellow eels (Figure 1). Out of these genes, 505 genes were higher in their transcript level and 141 genes were lower in their transcript level. An initial classification of differentially transcribed genes by Gene Ontology categories using DAVID revealed significant changes in 28 cellular processes in uninfected silver eels (*P* < 0.05), as compared with uninfected yellow eels (Table 2). Several of these categories were related to protein metabolism, translation, protein localization, and transport, but also to mitosis and cell cycle. Significantly affected were also the categories “oxidation reduction” and “ncRNA metabolic process” (Table 2).

**Table 2:** **Functional annotation analysis based on GO terms showing significantly affected cellular processes in eel gas gland tissue comparing uninfected yellow eels with uninfected silver eels (DAVID)**.

**Term**	**Count**	**%**	***P*-Value**
GO:0006793~phosphorus metabolic process	1123	6.461	0.010
GO:0006796~phosphate metabolic process	1122	6.455	0.010
GO:0006508~proteolysis	1057	6.081	0.020
GO:0008104~protein localization	970	5.581	0.003
GO:0016310~phosphorylation	929	5.345	0.024
GO:0045184~establishment of protein localization	855	4.919	0.003
GO:0015031~protein transport	848	4.879	0.003
GO:0007049~cell cycle	759	4.367	0.012
GO:0044257~cellular protein catabolic process	653	3.757	0.044
GO:0051603~proteolysis involved in cellular protein catabolic process	651	3.745	0.045
GO:0046907~intracellular transport	625	3.596	0.013
GO:0055114~oxidation reduction	619	3.561	0.015
GO:0019941~modification-dependent protein catabolic process	616	3.544	0.034
GO:0043632~modification-dependent macromolecule catabolic process	616	3.544	0.034
GO:0006396~RNA processing	547	3.147	0.006
GO:0012501~programmed cell death	536	3.084	0.045
GO:0006915~apoptosis	530	3.049	0.048
GO:0022402~cell cycle process	495	2.848	0.035
GO:0051276~chromosome organization	471	2.710	0.020
GO:0007010~cytoskeleton organization	442	2.543	0.031
GO:0022403~cell cycle phase	373	2.146	0.010
GO:0051301~cell division	344	1.979	0.004
GO:0006412~translation	338	1.945	0.004
GO:0016568~chromatin modification	337	1.939	0.020
GO:0000278~mitotic cell cycle	336	1.933	0.020
GO:0000279~M phase	309	1.778	0.032
GO:0008380~RNA splicing	283	1.628	0.013
GO:0034660~ncRNA metabolic process	229	1.318	0.037

### Transcriptional changes in eel swimbladder tissue related to silvering

To get more detailed insight into the affected metabolic pathways, genes with different mRNA expression level participating in defined pathways expected to be involved in swimbladder function and/or silvering were extracted from GO biological processes. In swimbladder tissue of silver eels, a number of genes involved in carbohydrate transport showed a higher mRNA expression level (Table 3). Interestingly, the mRNA level of monocarboxylate transporter 9 (mot9) was significantly reduced, and genes involved in glycolysis were not significantly modified in their expression level. Two members of the facilitated glucose transport proteins, however, were several-fold higher in their mRNA expression level, namely gtr5 and gtr6. In addition, tyrosin-protein-kinase fyn mRNA was largely elevated. Noteworthy is the almost 10-fold elevation of the carbonic anhydrase 12 (cah12) transcript, while cah6 mRNA was reduced.

**Table 3:** **Differentially transcribed genes based on GO terms glucose or energy metabolism in uninfected and in infected swimbladder silver eel swimbladder tissue as compared with uninfected yellow eel swimbladder tissue (*P* < 0.01)**.

**Gene**	**Name**	**Description**	**Fold change**	**Fold change**
			**Uninfected silver**	**Infected silver**
g27889	fyn	Tyrosine-protein kinase fyn	5.69	
g15190	cah6	Carbonic anhydrase 6	0.29	
g10376	wbs14	Williams-beuren syndr. chrom. region 14 protein	0.11	
g13449	gtr5	Facilitated glucose transporter member 5	82.99	31.03
g977	al1a3	Aldehyde dehydrogenase family 1 member a3	17.28	10.83
g13210	cah12	Carbonic anhydrase 12	9.20	5.46
g26078	cxcl2	c-x-c motif chemokine 2	8.67	7.37
g25279	gtr6	Facilitated glucose transporter member 6	6.26	5.80
g14711	ts101	Tumor susceptibility gene 101 protein	5.68	5.74
g1014	mot9	Monocarboxylate transporter 9	0.19	0.15
g18418	sca5a1	Sodium glucose transport1		44.42
g7770	mt12b	Monocarboxylate transporter 12-b		8.54
g3149	ednrb	Endothelin b receptor		8.02
g12175	enob	Beta-enolase		6.96
g12848	gtr3	Facilitated glucose transporter member 3		4.06
g22559	hxk2	Hexokinase-2		3.83
g8735	ssr1	Somatostatin receptor type 1		0.15

In uninfected silver eel swimbladder, 28 genes connected to the metabolism of reactive oxygen species (ROS) were affected (Table 4). The mRNA species of these ROS related genes showing the largest elevation in uninfected silver eels was nadh dehydrogenase 1 (nqo1; 98-fold elevated). Among the additionally elevated genes were also cytochrome p450 1b1 (cp1b1), neuronal pentraxin (nptx1) ribonucleoside-diphosphate reductase (rir2), and glutathione peroxidase (gpx7).

**Table 4:** **Differentially transcribed genes based on GO terms response to oxidative stress and redox homeostasis in uninfected and in infected swimbladder silver eel swimbladder tissue as compared with uninfected yellow eel swimbladder tissue (*P* < 0.01)**.

**Gene**	**Name**	**Description**	**Fold change**	**Fold change**
			**Uninfected silver**	**Infected silver**
g9710	nqo1	Nad h dehydrogenase 1	98.49	
g26398	nptx1	Neuronal pentraxin-1	19.97	
g5410	hspbb	Heat shock protein beta-11	18.24	
g14663	cp1b1	Cytochrome p450 1b1	11.19	
g24694	mmp17	Matrix metalloproteinase-17	8.09	
g20370	gpx7	Glutathione peroxidase 7	7.44	
g13550	dus5	Dual specificity protein phosphatase 5	7.23	
g19822	co5a1	Collagen alpha-1 chain flags: precursor	3.95	
g11852	hfe	Hereditary hemochromatosis protein	0.55	
g13602	angp1	Angiopoietin-1	0.17	
g38375	opla	5-oxoprolinase	0.16	
g27278	opla	5-oxoprolinase	0.09	
g43641	opla	5-oxoprolinase	0.08	
g12711	mmp9	Matrix metalloproteinase-9	0.08	
g26738	hfe	Hereditary hemochromatosis protein	0.04	
g12220	rir2	Ribonucleoside-diphosphate reductase subunit m2	18.90	16.21
g6958	stat1	Signal transducer and activator of transcription 1	13.70	11.91
g26186	sh3l3	Sh3 domain-binding glutamic acid-rich-like protein 3	12.98	15.69
g28565	rir2	Ribonucleoside-diphosphate reductase subunit m2	8.45	12.33
g13141	bcar3	Breast cancer anti-estrogen resistance protein 3	6.12	6.48
g41989	cflar	Casp8 and fadd-like apoptosis regulator	5.31	5.28
g1952	klf4	Krueppel-like factor 4	0.24	0.22
g20403	klf4	Krueppel-like factor 4	0.20	0.19
g3963	ncam2	Neural cell adhesion molecule 2	0.20	0.26
g9277	jun	Transcription factor ap-1	0.10	0.13
g12409	fos	Proto-oncogene c-fos	0.08	0.21
g3322	fos	Proto-oncogene c-fos	0.08	0.07
g11898	fosb	Protein fosb	0.05	0.08
g12274	mfha1	Malignant fibrous histiocytoma-amplified sequence 1		Inf
g21373	atty	Tyrosine aminotransferase		Inf
g6637	cy24b	Cytochrome b-245 heavy chain		27.50
g7735	cy24b	Cytochrome b-245 heavy chain		5.67
g7816	hmox	Heme oxygenase		5.53
g9598	pxdn	Peroxidasin homolog flags: precursor		4.47
g8735	ssr1	Somatostatin receptor type 1		0.15
g2030	pa24c	Cytosolic phospholipase a2 gamma		0.15
g24134	stx2	Syntaxin-2		0.05

An improvement in swimbladder function could also be achieved by a reduction in gas permeability. Therefore, genes associated to the extracellular matrix were analyzed (Table 5). Nine genes were affected in uninfected silver eels, and only two of these showed a lower steady state mRNA level. Among the genes elevated in their transcription level were mimecan (mime), collagen alpha (co5a1), collagenase 3 (mmp13), and matrix metalloproteinase-17 (mmp17), a lower level was detected for neural adhesion molecule 2 (ncam2) and metalloproteinase-9 (mmp9).

**Table 5:** **Differentially transcribed genes based on GO terms extracellular matrix constituents in uninfected and in infected swimbladder silver eel swimbladder tissue as compared with uninfected yellow eel swimbladder tissue (*P* < 0.01)**.

**Gene**	**Name**	**Description**	**Fold change**	**Fold change**
			**Uninfected silver**	**Infected silver**
g23807	mmp13	Collagenase3	Inf	
g13732	mime	Mimecan	12.51	
g24694	mmp17	Matrix metalloproteinase-17	8.09	
g19822	co5a1	Collagen alpha-1 chain	3.95	
g12711	mmp9	Matrix metalloproteinase-9	0.08	
g7750	tsp4b	Thrombospondin-4-b	15.23	27.87
g12933	scub1	Signal cub and egf-like domain-cont prot 1	11.61	28.16
g22618	chia	Acidic mammalian chitinase	3.84	13.40
g3963	ncam2	Neural cell adhesion molecule 2	0.20	0.26
g24192	muc5b	Mucin-5b		Inf
g35363	muc5a	Mucin-5ac		Inf
g37402	muc5b	Mucin-5b		Inf
g28800	muc5b	Mucin-5b		38.64
g34568	muc5a	Mucin-5ac		38.60
g41486	muc5a	Mucin-5ac		31.98
g18964	muc5a	Mucin-5ac		24.35
g23617	chia	Acidic mammalian chitinase		14.90
g15581	tecta	Alpha-tectorin		4.46

Looking at genes associated with ion exchange, 28 genes were differentially expressed in uninfected eel swimbladder, out of which 12 were higher in their expression level (Supplementary Table 1). Remarkable was the elevation of NADH dehydrogenase and of sodium potassium-transporting atpase (atng). In addition, a potassium channel was elevated (kcnb1), while cystic fibrosis transmembrane conductance channel (cftr), and sodium hydrogen exchanger 1 (sl9a1) were reduced in their mRNA expression level.

An increased capillarization has been described for silver eel swimbladder, therefore the transcriptional changes observed in angiogenesis related genes were addressed (Table 6). In uninfected silver eel swimbladder tissue, mRNAs levels of hormones involved in angiogenesis like VEGF and angiopoietin (vegfc; angp1) were reduced, and several inhibitors of angiogenesis were elevated (cytc; cxl10; scub1; tsp4b; sem3f).

**Table 6:** **Differentially transcribed genes based on GO terms angiogenesis or vasculogenesis in uninfected and in infected swimbladder silver eel swimbladder tissue as compared with uninfected yellow eel swimbladder tissue (*P* < 0.01)**.

**Gene**	**Name**	**Description**	**Fold change**	**Fold change**
			**Uninfected silver**	**Infected silver**
g21158	cytc	Cystatin-c	Inf	
g35655	cxl10	c-x-c motif chemokine 10	12.82	
g28118	scub1	Signal cub and egf-like domain-cont prot 1 flags	11.63	
g24694	mmp17	Matrix metalloproteinase-17	8.09	
g8995	scub2	Signal cub and egf-like domain-cont prot 2	7.87	
g36354	par14	Poly polymerase 14	4.77	
g3471	tnni2	Troponin fast skeletal muscle	4.35	
g19822	co5a1	Collagen alpha-1 chain flags: precursor	3.95	
g3852	vegfc	Vascular endothelial growth factor c	0.24	
g13602	angp1	Angiopoietin-1	0.17	
g26552	robo2	Roundabout homolog 2 flags: precursor	0.14	
g20875	pf2r	Prostaglandin f2-alpha receptor	0.13	
g7112	foxg1	Forkhead box protein g1	0.09	
g31917	ita10	Integrin alpha-10 flags: precursor	0.04	
g49	sem3f	Semaphorin-3f	Inf	Inf
g15237	par12	Poly polymerase 12	45.11	31.02
g18110	par11	Poly polymerase 11	44.06	34.90
g17733	fhr2	Complement factor h-related protein 2	33.65	6.52
g977	al1a3	Aldehyde dehydrogenase family 1 member a3	17.28	10.83
g7750	tsp4b	Thrombospondin-4-b	15.23	27.87
g12933	scub1	Signal cub and egf-like domain-cont prot 1 flags	11.61	28.16
g38983	par12	Poly polymerase 12	11.58	8.08
g11223	lyve1	Lymph vessel endoth hyaluronic acid receptor 1	10.63	12.21
g5447	socs1	Suppressor of cytokine signaling 1	9.60	5.04
g13141	bcar3	Breast cancer anti-estrogen resistance protein 3	6.12	6.48
g41989	cflar	Casp8 and fadd-like apoptosis regulator	5.31	5.28
g9669	bmp1	Bone morphogenetic protein 1	4.14	7.27
g1952	klf4	Krueppel-like factor 4	0.24	0.22
g20403	klf4	Krueppel-like factor 4	0.20	0.19
g9277	jun	Transcription factor ap-1	0.10	0.13
g13534	cxd2	Gap junction delta-2 protein	0.07	0.12
g22125	twhh	Tiggy-winkle hedgehog protein		Inf
g2293	zc12c	Probable ribonuclease zc3h12c		Inf
g513	sem3c	Semaphorin-3c		Inf
g6564	prg4	Proteoglycan 4		73.72
g6637	cy24b	Cytochrome b-245 heavy chain		27.50
g12811	co3	Complement c3 contains:		20.61
g16076	apoh	Beta-2-glycoprotein 1		13.73
g20378	trhde	Thyrotropin-releasing hormone-degrading ectoenzyme		9.75
g3149	ednrb	Endothelin b receptor		8.02
g5296	cn073	Sec6-like protein c14orf73		7.41
g36054	s1pr4	Sphingosine 1-phosphate receptor 4		6.79
g7735	cy24b	Cytochrome b-245 heavy chain		5.67
g7816	hmox	Heme oxygenase		5.53
g29481	mk11	Mitogen-activated protein kinase 11		5.51
g15158	apj	Apelin receptor		4.38
g44679	co5	Complement c5		4.37
g7116	c5ar	C5a anaphylatoxin chemotactic receptor		4.32
g12898	ptprh	Receptor-type tyrosine-protein phosphatase h		4.15
g26433	ccr4	C-c chemokine receptor type 4		4.00
g20847	myo1f	Myosin-if		3.53
g16879	tmps6	Transmembrane protease serine 6		0.18
g8735	ssr1	Somatostatin receptor type 1		0.15
g15224	tbx18	T-box transcription factor tbx18		0.00

Differentially transcribed immune related genes were also extracted from GO biological process. One hundred and twenty four genes were differentially transcribed in uninfected silver eel swimbladder, and only 16 out of these genes were reduced in their transcript level (Supplementary Table 2). Many of these genes were more than 10-fold elevated. Among the genes with reduced mRNA expression level were two integrins (ita2, ita10), showing a 14- and 28-fold lower expression level, respectively. Among the elevated genes were several complement proteins, stat1 and interferon induced genes [e.g., 6 mx genes (18-94-fold), 3 IF44 genes (15-23-fold), 2 gvin1 genes (eight-fold)] and interferon regulatory factors, immunoglobulin light chain, and neuronal pentraxin-1 (stat1; cfab; c1qc; in35; iigp5; nptx1).

Surprising was the large number of genes related to sexual maturation that were significantly modified in their transcription level in swimbladder tissue (Supplementary Table 3). The transcription level of 68 genes was modified in uninfected silver eel swimbladder. Among these were three zona pellucida genes, which were elevated about 200-fold (zp1, zp2, and also three copies of zp3).

### Transcription changes in infected silver eel swimbladder tissue

To assess the influence of an *A. crassus* infection on the silvering related changes in swimbladder tissue we compared these silvering induced changes in the level of specific transcripts with changes observed in infected silver eel swimbladder tissue if compared with uninfected yellow eel swimbladder tissue. Compared with uninfected yellow eels in infected silver eels 782 genes were different in their mRNA expression level at the significance level of *P* < 0.01 (Figure 1). Of these genes, 602 were elevated, and 180 reduced. A comparison of the modified genes between uninfected yellow and silver eels and uninfected yellow and infected silver eels revealed that more than 50% of the genes that were differentially transcribed in infected silver eels (395 genes) were not affected in uninfected swimbladder tissue (Figure 1). The difference became even more obvious when looking at the genes with reduced steady state mRNA level. Of the 180 genes with reduced expression level in infected silver eel swimbladder tissue only 42% were also reduced in uninfected swimbladder tissue. Accordingly, although the total number of genes affected was similar in uninfected and infected silver eels, quite different sets of genes were affected in these two experimental groups. In none of the genes with different mRNA expression level in the two experimental groups opposite changes were detected.

Gene Ontology category analysis, performed using DAVID revealed that in infected silver eels the number of significantly modified functional categories was reduced from 28 to 17 (Table 7); and 12 of these categories were also significantly affected in uninfected silver eels. Functional categories expressed in uninfected silver eels but not in infected silver eels included, for example, “intracellular transport,” “cell cycle process,” “cell division,” “programmed cell death” (“apoptosis”), and “ncRNA metabolic process.” In turn, exclusively in infected silver eels significantly affected functional categories included “chromatin organization,” “response to DNA damage stimulus,” and “cellular response to stress.”

**Table 7:** **Functional annotation analysis based on GO terms showing significantly affected cellular processes in eel gas gland tissue comparing uninfected yellow eels with infected silver eels (DAVID)**.

**Term**	**Count**	**%**	***P*-Value**
GO:0006793~phosphorus metabolic process	1130	6.445	0.004
GO:0006796~phosphate metabolic process	1129	6.439	0.004
GO:0008104~protein localization	974	5.555	0.008
GO:0016310~phosphorylation	936	5.338	0.004
GO:0045184~establishment of protein localization	858	4.893	0.010
GO:0015031~protein transport	851	4.853	0.011
^*^GO:0006468~protein amino acid phosphorylation	835	4.762	0.012
^*^GO:0009057~macromolecule catabolic process	794	4.528	0.043
GO:0007049~cell cycle	764	4.357	0.003
GO:0055114~oxidation reduction	624	3.559	0.001
^*^GO:0033554~cellular response to stress	528	3.011	0.014
GO:0051276~chromosome organization	474	2.703	0.006
^*^GO:0006325~chromatin organization	385	2.196	0.020
^*^GO:0006974~response to DNA damage stimulus	356	2.030	0.029
GO:0006412~translation	338	1.928	0.037
GO:0016568~chromatin modification	338	1.928	0.037
GO:0000278~mitotic cell cycle	337	1.922	0.038

* *indicates categories significantly different only in infected silver eels*.

Table 8 shows an overview of changes in the transcription level detected in specific pathways that were—based on our previous experimental studies—expected to be involved in swimbladder function and/or silvering. The most obvious differences in the comparison of uninfected yellow eels with either uninfected silver eel or infected silver eels were observed in the transcription level of genes related to ion transport and extracellular matrix. In both groups < 25% of the genes showing a modified mRNA expression level were affected in both groups, meaning that more than 75% of the genes were affected either only in uninfected silver eel swimbladder tissue, or in infected eel swimbladder tissue. A higher overlap (above 40%) was observed in the transcription changes of genes related to glucose metabolism, immune response, and sexual maturation.

**Table 8:** **Overview of the pathways analyzed (Tables 3–6 and Supplementary Tables 1–3) and the total number of genes affected in both experimental groups**.

**GO term**	**No**.	**Uninf. (%)**	**Inf. (%)**	**Common (%)**	**Up**	**Down**	**Up**	**Down**
					**Uninfected**		**Infected**	
Ion exchange	52	33	46	21	13	15	20	15
ROS metabolism	37	41	24	35	14	14	12	10
Maturation	96	28	29	43	50	15	46	23
Immune response	200	21	38	41	108	16	140	18
Glucose metabolism	17	18	41	41	7	3	12	2
Extracellular matrix	18	28	50	22	7	2	12	1
Angiogenesis	54	26	43	31	21	10	33	7

*The table also shows the percentage of genes affected only in uninfected or in infected swimbladder tissue, and the percentage of genes affected in both groups. In addition, the total number of up and down-regulated genes in uninfected and infected silver eels as compared with uninfected yellow eels is shown*.

A detailed analysis of the pathways revealed that in contrast to uninfected silver eels in infected silver eel swimbladder glycolytic genes (enob, hxk2) were significantly elevated in their mRNA expression level (Table 3). Monocarboxylate transporter 9 (mot9) was reduced, as also seen in uninfected silver eels, while monocarboxylate transporter 12-b (mt12b) was 8.5-fold elevated. In infected swimbladders, a higher transcript level was also detected for glucose transporters (gtr3, 5, 6). Particularly noteworthy was the 44-fold elevation of the sodium dependent glucose co-transporter (sca5a1).

In infected silver eel swimbladder tissue, 22 genes connected to the metabolism of ROS were affected (Table 4), out of which 13 genes were also differentially transcribed in uninfected silver eels. Remarkable was that in contrast to uninfected silver eels glutathione peroxidase (gpx7) was not affected. Among the additionally elevated genes in infected silver eels was ribonucleoside-diphosphate reductase (rir2). Only in infected silver eels, heme oxygenase (hmox), and cytochrome b-245-heavy chain (cy24b) showed an elevated steady state mRNA level.

In infected silver eel swimbladder, 13 genes associated to the extracellular matrix were differentially transcribed, and only one of them was reduced in mRNA expression (Table 5). In contrast to uninfected silver eels, in infected eels several mucin genes were more than 20-fold elevated or even switched on (muc5a, muc5b, muc5ac). In fact, seven out of nine genes with an elevated transcript level in infected swimbladder tissue were mucin genes.

Looking at genes associated with ion exchange, 35 genes were differentially transcribed in infected silver eel swimbladder, out of which 11 were also affected in uninfected eel swimbladder (Supplementary Table 1). Sodium potassium transporting ATPase subunit g1 (at1b2) was more than seven-fold reduced, while, v-type proton ATPase subunit g1 (vatg1) was four-fold upregulated. Almost 10-fold elevated was also metalloreductase steap4 (stea4).

In infected eels, the down-regulation of the angiogenesis stimulating hormones was not observed, they remained unchanged in transcription levels. Most of the affected genes were upregulated (32), while only seven genes were down-regulated. Among the upregulated enzymes were thyrotropin-releasing hormone-degrading ectoenzyme (trhde) and MAP kinase 11 (mk11).

As to be expected in infected silver eel swimbladder tissue, 158 immune related genes were modified in the transcription level, and 76 of these genes were selectively affected in infected eel swimbladder tissue, and only nine of these genes showed a reduced mRNA level (Supplementary Table 2).

Surprising was the large number of genes related to sexual maturation that were significantly modified in their transcription level in infected eel swimbladder tissue (Supplementary Table 3). The transcription level of 69 genes was modified in infected silver eel swimbladder, and 41 of these genes were affected in both, infected and uninfected silver eels as compared with uninfected yellow eels.

## Discussion

### Transcription changes connected to silvering

Silvering is connected to large scale transcriptional changes in swimbladder tissue, and 78% of the affected genes were elevated in their transcriptional level. A difference in steady state mRNA level can be achieved either by increasing the rate of transcription, or by decreasing the rate of mRNA break down. Thus, although we measured steady state levels of mRNA transcripts, changes in these mRNA levels were based on changes in transcription.

Cellular processes, affected by changes in steady state mRNA levels, were connected to cell cycle, cell division, and programmed cell death. In addition, a large number of genes involved in protein metabolism and transport have been modified in their transcription level. Assuming that these changes are at least partially translated into changes in translational activity (i.e., into protein synthesis), our data suggested that silvering was connected to a significant protein and cell turn-over in gas gland tissue. Intestinal epithelia are known for their high turn-over rate, and swimbladder tissue is derived from the foregut. Tissue renewal especially in connection to the silvering process, preparing the swimbladder for the spawning migration, appears quite possible.

Transcription changes observed for genes involved in the formation of the extracellular matrix supported this conclusion. In silver eel swimbladder tissue, several genes related to the formation and renewal of the extracellular matrix showed an elevated transcript level. This could result in an improvement of gas impermeability, which has been reported to occur during silvering for the American eel and the Japanese eel (Kleckner, 1980a,b; Yamada et al., 2001).

Gas secretion in the swimbladder is dependent on glucose metabolism, resulting in the production and release of lactic acid and of CO_2_, generated in the pentose phosphate shunt (Pelster and Scheid, 1993; Walsh and Milligan, 1993; Pelster et al., 1994; Pelster, 1995b). In uninfected swimbladder tissue, surprisingly, no significant change in the mRNA level of glycolytic enzymes was detected, while two members of the facilitated glucose transporters (gtr5, 6) were several-fold elevated. This suggested that metabolic activity of gas gland tissue is controlled by glucose uptake into the cell. Production of CO_2_ in the pentose phosphate shunt, subsequent release into the blood stream, and countercurrent concentration of CO_2_ have been identified as crucial steps in the initiation of gas secretion (Kobayashi et al., 1990; Pelster et al., 1994). Carbonic anhydrase activity including a membrane bound carbonic anhydrase (Pelster, 1995a; Würtz et al., 1999) has been shown to enhance CO_2_ movements in swimbladder tissue of the European eel, and the membrane bound carbonic anhydrase12 (Supuran, 2008) was nine-fold elevated in the mRNA level. The data therefore suggest that during silvering the pentose phosphate shunt gained importance with the production of CO_2_ for gas secretion. Membrane bound carbonic anhydrase facilitates the movements of CO_2_, resulting also in an acidification of blood and switching on of the Root effect (Pelster and Weber, 1991; Pelster and Randall, 1998), facilitating oxygen secretion into the swimbladder. It appears possible that during vertical migrations in the ocean glycolytic activity and lactate production may be enhanced as a second step, following the initiation of gas secretion via CO_2_ production in the pentose phosphate shunt.

Considering ion transport, silvering appeared to result in an enhancement of acid secretion. The mRNA level of Na^+^/H^+^ exchange regulatory cofactor (nhrf3) was 110-fold elevated, suggesting a stimulation of acid release via Na^+^/H^+^ exchange. Involvement of Na^+^/H^+^ exchange in acid secretion of gas gland cells has been shown previously (Pelster, 1995a). An increased Na^+^/H^+^ exchange activity requires an enhanced Na^+^/K^+^-ATPase activity in order to retain the sodium gradient, and Na^+^/K^+^-ATPase subunit gamma mRNA was indeed elevated. Overall, in silver eel swimbladder more ion transport protein coding genes were significantly reduced in their transcript level than elevated.

To assure appropriate gas secretion, an adequate capillarization and blood supply to the gas gland tissue must be established, but our data did not provide evidence for an improved capillarization of swimbladder tissue. mRNA levels of hormones typically involved in angiogenesis (angiopoietin-1; vascular endothelial growth factor c) were even reduced in uninfected silver eels, and inhibitors of angiogenesis showed increased transcript levels. On the other hand, Kleckner (1980a) reported an increase in the length of rete mirabile capillaries for the American eel. It therefore may be concluded that capillarization of swimbladder tissue itself is not modified during silvering in the European eel, but the capacity of the rete mirabile for countercurrent multiplication is improved by increasing the rete length in order to achieve higher oxygen partial pressures in the swimbladder (Pelster, 1997, 2013).

Due to the high oxygen partial pressures to be expected in the eel swimbladder during the spawning migration, we expected that the ROS defense pathways would be enhanced. Indeed, the results showed 28 genes with a modified transcript level in uninfected silver eel swimbladder tissue, and many of these genes were elevated. Of particular importance appeared to be the almost 100-fold elevation of NADH dehydrogenase, controlling the ratio between NADH and NAD^+^. Noteworthy was also the elevation of glutathione peroxidase, one of the main enzymes involved in the breakdown of ROS. In a recent study we could show that the concentration of total glutathione (GSH + GSSG) is significantly elevated in silver eel swimbladder tissue as compared with yellow eel swimbladder (Schneebauer et al., 2016). The reduction of oxidized glutathione is dependent on the presence of NADPH, and the pentose phosphate shunt, in addition to the production of CO_2_ (see above), generates NADPH. Taken together these results supported the conclusion that the glutathione-based ROS defense is of increasing importance in silver eels.

Somewhat surprising was that in uninfected swimbladder tissue a large number of immune related genes was elevated in the steady state mRNA level. With the changes from freshwater to seawater, eels are exposed to a different set of pathogens. It therefore appears plausible that the silvering process includes a change in the transcription pattern of immune related genes in preparation for the new environment. Another possibility would be that Dutch silver eels from the IJsselmeer are exposed to a different set of pathogens, not encountered by yellow eels in Lake Constance. In this case, the immune response might not be specifically connected to the process of silvering, but simply the results of a different environment. On the other hand, trying to identify marker genes of pectoral fin development a large number of differentially expressed immune related genes has also been detected in the European eel (Dirks et al., 2014). The immune system appears to be quite responsive to environmental changes.

Yellow eels are immature and the silvering process has always been seen in the context of maturation, although sexual maturity and gonadal development probably is completed only at the time of arrival at the Sargasso Sea (Dufour et al., 2003). Unexpected, however, was to see that a large number of maturation related genes was significantly modified in the transcription level in swimbladder tissue, which has nothing to do with reproduction. The swimbladder is an isolated tissue in the abdominal cavity of a fish, and after careful dissection and rinsing of the tissue contamination from other tissues could be excluded, except for some blood, which always will be present in dissected tissues. We therefore conclude that the changes in the mRNA level observed in our samples reflect changes in swimbladder tissue.

Particularly puzzling was the almost 200-fold elevation of three different zona pellucida genes in two out of five uninfected silver eel swimbladder tissue samples. Zona pellucida proteins are found in the extracellular matrix surrounding the egg, the so-called chorion. Expression of zona pellucida genes has been described in fish (Wang and Gong, 1999; Conner and Hughes, 2003), but so far at least ZP2 and ZP3 expression has been reported to be restricted to ovary tissue. ZP1, however, has been found in liver tissue (Conner and Hughes, 2003) or in a number other tissues including spleen and kidney (Chuang-Ju et al., 2011). The physiological meaning of transcription of all three genes in uninfected silver eel swimbladder tissue remained elusive. The severe elevation of these mRNA species in uninfected silver eels clearly suggested, however, that the silvering was connected to the onset of sexual maturation. A possible explanation for these changes in maturation related gene expression in swimbladder tissue could be that the maturation process of the gonads induced expression changes in other organs, including organs not immediately connected to sexual maturation. During maturation, an elevation in plasma steroid concentration has been reported (Burgerhout et al., 2016), and these hormones may of course induce expression changes in other tissues as well.

### Transcriptional changes observed in infected eel swimbladder tissue

At first glance, the number of genes showing a different expression level and the number of genes showing a higher mRNA level in infected silver eels was somewhat similar to the results obtained for uninfected silver eels. A detailed analysis at the level of affected biological processes, however, revealed significant differences in the steady state mRNA expression level between the two groups. If these differences in mRNA level are at least partially translated into changes in protein concentration the silvering process may be significantly modified by the infection of the swimbladder with the nematode *A. crassus*.

In contrast to uninfected silver eels, in infected silver eel swimbladder tissue the GO terms “apoptosis” and “programmed cell death” were not significantly affected, and only very few GO terms related to protein metabolism or transport and to the cell cycle were significantly different. This suggested that the infection of the swimbladder reduced the tissue renewal, typically taking place during silvering. Instead, the GO terms “cellular response to stress” and “response to DNA damage” were affected. The feeding activity of the nematode causes tissue damage (Molnár et al., 1993; Beregi et al., 1998; Würtz and Taraschewski, 2000; Lefebvre et al., 2011, 2013), thereby activating genes involved in stress response and tissue repair. This finally results in a severe thickening of swimbladder tissue, and the unicellular gas gland tissue is transformed to a hyperplastic multicellular epithelium (Molnár et al., 1995; Nimeth et al., 2000; Würtz and Taraschewski, 2000).

The defense reaction of the European eel became also obvious when looking at the transcript levels of immune related genes and of extracellular matrix related genes. While in uninfected eel swimbladder tissue, various genes related to the formation and restructuring of the extracellular matrix were almost exclusively elevated, in infected swimbladder tissue various mucin genes were elevated. Cutaneous mucus is considered the first line of defense against infection (Esteban, 2012). The induction and/or the between 20- and 40-fold elevation of seven mucin genes only in infected swimbladder tissue therefore appeared to be a clear defense reaction of the host tissue. A strong defense reaction was also visible in the transcript level changes observed in immune related genes. In infected eel swimbladder tissue, 158 immune related genes were different in their mRNA level compared with uninfected yellow eels, and only 18 of these genes were reduced. More than 40% of these mRNA levels were modified exclusively in infected swimbladder tissue. Activation of the immune system has previously been indicated by presence of macrophages in infected tissue (Beregi et al., 1998; Würtz and Taraschewski, 2000). The significant elevation of the mRNA level of a number of complement proteins, of various interferon inducible genes, for example, and of several members of tripartite motive family of proteins, also inducible by interferons, supported the conclusion that the infection of the swimbladder with nematodes provoked a strong response of the immune system in the eel.

An increased mRNA level of glycolytic genes was detected in infected swimbladder tissue, including in particular hexokinase2, contributing to an initial phosphorylation of glucose taken up into the cells. In addition, in infected swimbladder tissue mRNA levels of genes encoding glucose transport proteins including a sodium dependent glucose transporter were significantly elevated. Infected swimbladder tissue becomes a multilayered epithelium (see above), and diffusion distances for the supply are significantly enlarged. Although in infected swimbladder a down-regulation of angiopoietic hormones was not observed, no evidence for an upregulation of angiopoietic factors was detected. In addition, the oxygen content of a heavily infected swimbladder is significantly lower than of an uninfected swimbladder (Würtz et al., 1996). Taken together, these data therefore suggest that oxygen supply to the thickened tissue became limiting and therefore anaerobic glycolysis with the formation of lactic acid was enhanced, increasing the demand for glucose in swimbladder tissue. Because the nematode is histophagous (Fazio et al., 2008), a proper nutrient supply to the swimbladder tissue is certainly also beneficial for the parasite. The elevated transcript level of genes encoding glucose transporters therefore may well have been for the benefit of the parasite.

These considerations raise the question, whether the nematode is able to affect the activity and gene expression in swimbladder tissue. A strong hint for this would be, if the expression of certain genes would be reversed in infected tissue as compared with uninfected tissue. At the significance level tested, this was not observed. On the other hand, the enhanced expression of heme oxygenase (hmox) and of metalloreductase (stea4) exclusively in infected tissue could indeed have been induced by the parasite. Heme oxygenase breaks down the heme of hemoglobin, resulting in Fe^3+^, which can be reduced to Fe^2+^ by metalloreductase. Previous studies indicated that the parasite may stimulate hematopoiesis by enhancing expression of the hemoglobin α-chain (Fazio et al., 2009). This could indicate that the parasite stimulated hemoglobin synthesis and subsequent degradation so that it can easily feed on these break down products.

Remarkable in this context was also the reduced level of Na^+^/K^+^-ATPase subunit beta2 (at1b2) mRNA, while in uninfected silver eels another subunit of this ATPase was elevated (see above). While in uninfected eels most of the mRNA species encoding genes involved in ion transport were reduced, in infected eels the majority was elevated. Accordingly, in terms of ion transport only 21% of the affected mRNA species were affected in uninfected as well as in infected silver eels, which was the lowest number of expression overlap. This observation underlined the severe impact of the parasite on swimbladder function.

Elevation of mRNA species of a number of ROS related genes has also been observed in infected swimbladder tissues, suggesting that the infection of the swimbladder increased the level of oxidative stress in this organ in spite of the fact, that the oxygen content of an infected swimbladder is significantly lower (Würtz et al., 1996). In infected silver eels, however, glutathione peroxidase, a crucial enzyme in ROS defense elevated in uninfected silver eels, was not elevated.

## Perspectives

Our Illumina sequencing data revealed large scale changes in the steady state mRNA level in swimbladder tissue during silvering in the European eel. These changes suggest that swimbladder gas permeability and ROS defense systems are improved during silvering, and that the production and diffusion of CO_2_ are of particular importance. The data suggest that glucose metabolism in swimbladder tissue is mainly regulated by glucose uptake of the cells. While these data clearly support the notion that the swimbladder is of importance during spawning migration, a theoretical analysis revealed that swimbladder volume can hardly be adjusted to retain the status of neutral buoyancy during vertical migrations. Keeping swimbladder gas content constant in the face of changing hydrostatic pressure, however, significantly increases energy expenditure for swimming (Pelster, 2015). The actual contribution of the swimbladder to the spawning migration therefore remains open for further analysis. Analysis of swimbladder function under changing hydrostatic pressure probably would be very helpful to answer this question. Furthermore, the changes in the mRNA level seen in a large number of genes related to sexual maturation during silvering suggests that these studies could also be very helpful to elucidate the molecular and physiological changes connected to sexual maturation.

The data also show that an infection of the swimbladder with the nematode *A. crassus* significantly impairs the molecular changes observed in swimbladder tissue during silvering. Eel tissue showed a strong immune response, metabolic adjustments seen in uninfected tissue were largely abolished and the modification of extracellular matrix components was completely different. These altered cellular processes could significantly contribute to the detrimental changes in swimbladder function in infected eels as observed in a previous study (Würtz et al., 1996). This may significantly compromise a successful spawning migration. Further development of the infection status of the European eel therefore may be crucial for the development of the population.

## Author contributions

BP—designed the study, analyzed data, wrote the first draft of the paper. GS—collected tissues, analyzed the data, contributed to writing the paper. RD—collected tissues, performed illumina seq, performed original data alinement and analysis, contributed to writing the paper.

### Conflict of interest statement

The authors declare that the research was conducted in the absence of any commercial or financial relationships that could be construed as a potential conflict of interest.

## References

[B1] Aarestrup K. Okland F. Hansen M. M. Righton D. Gargan P. Castonguay M.. (2009). Oceanic spawning migration of the european eel (*Anguilla anguilla*). Science 325, 1660. 10.1126/science.1178120 19779192

[B2] Als T. D. Hansen M. M. Maes G. E. Castonguay M. Riemann L. Aarestrup K.. (2011). All roads lead to home: panmixia of European eel in the Sargasso Sea. Mol. Ecol. 20, 1333–1346. 10.1111/j.1365-294X.2011.05011.x 21299662

[B3] Anders S. Huber W. (2010). Differential expression analysis for sequence count data. Genome Biol. 11:R106. 10.1186/gb-2010-11-10-r106 20979621 PMC3218662

[B4] Anders S. Pyl P. T. Huber W. (2015). HTSeq - a Python framework to work with high-throughput sequencing data. Bioinformatics 31, 166–169. 10.1093/bioinformatics/btu638 25260700 PMC4287950

[B5] Beregi A. Molnár K. Békési L. Székely C. S. (1998). Radiodiagnostic method for studying swimbladder inflammation caused by *Anguillicola crassus* (Nematoda: Dracunculoidea). Dis. Aquat. Org. 34, 155–160. 10.3354/dao034155 9828409

[B6] Burgerhout E. Minegishi Y. Brittijn S. A. de Wijze D. L. Henkel C. V. Jansen H. J.. (2016). Changes in ovarian gene expression profiles and plasma hormone levels in maturing European eel (*Anguilla anguilla*); Biomarkers for broodstock selection. Gen. Comp. Endocrinol. 225, 185–196. 10.1016/j.ygcen.2015.08.006 26255685

[B7] Chuang-Ju L. Qi-Wei W. Xi-Hua C. Li Z. Hong C. Fang G.. (2011). Molecular characterization and expression pattern of three zona pellucida 3 genes in the Chinese sturgeon, Acipenser sinensis. Fish Physiol. Biochem. 37, 471–484. 10.1007/s10695-010-9448-x 21072685

[B8] Conner S. J. Hughes D. C. (2003). Analysis of fish ZP1/ZPB homologous genes–evidence for both genome duplication and species-specific amplification models of evolution. Reproduction 126, 347–352. 10.1530/rep.0.1260347 12968942

[B9] Dirks R. P. Burgerhout E. Brittijn S. A. de Wijze D. L. Ozupek H. Tuinhof-Koelma N.. (2014). Identification of molecular markers in pectoral fin to predict artificial maturation of female European eels (*Anguilla anguilla*). Gen. Comp. Endocrin. 204, 267–276. 10.1016/j.ygcen.2014.06.023 24992558

[B10] Dorn E. (1961). Über den feinbau der schwimmblase von *Anguilla vulgaris* L. Licht- und elektronenmikroskopische untersuchungen. Z. Zellforsch. 55, 849–912. 10.1007/BF00381654 13887393

[B11] Dufour S. Burzawa-Gerard E. Le Belle N. Sbaihi M. Vidal B. (2003). Reproductive endocrinology of the European eel, *Anguilla anguilla*, in Eel Biology, eds Aida K. Sukamoto K. T. Yamauchi K. (Tokyo: Springer), 373–383.

[B12] Durif C. Dufour S. Elie P. (2005). The silvering process of *Anguilla anguilla*: a new classification from the yellow resident to the silver migrating stage. J. Fish Biol. 66, 1025–1043. 10.1111/j.0022-1112.2005.00662.x

[B13] Esteban M. Á. (2012). An overview of the immunological defenses in fish skin. ISRN Immunol. 2012:853470 10.5402/2012/853470

[B14] Fazio G. Monée H. Da Silva C. Simon-Levert G. Allienne J. F. Lecomte-Finiger R.. (2009). Changes in gene expression in European eels (*Anguilla anguilla*) induced by infection with swim bladder nematodes (*Anguillicola crassus*). J. Parasitol. 95, 808–816. 10.1645/GE-1705.1 20049987

[B15] Fazio G. Moné H. Lecomte-Finiger R. Sasal P. (2008). Differential gene expression analysis in European eels *(Anguilla anguilla*, L. 1758) naturally infected by macroparasites. J. Parasitol. 94, 571–577. 10.1645/GE-1316.1 18605780

[B16] Fazio G. Sasal P. Mouahid G. Lecomte-Finiger R. Mone H. (2012). Swim bladder nematodes (*Anguillicoloides crassus*) disturb silvering in European eels (*Anguilla anguilla*). J. Parasitol. 98, 695–705. 10.1645/GE-2700.1 22404329

[B17] Henkel C. V. Burgerhout E. de Wijze D. L. Dirks R. P. Minegishi Y. Jansen H. J.. (2012). Primitive duplicate hox clusters in the European eel's genome. PLoS ONE 7:e32231. 10.1371/journal.pone.0032231 22384188 PMC3286462

[B18] Jacobsen M. W. Pujolar J. M. Bernatchez L. Munch K. Jian J. Niu Y.. (2014). Genomic footprints of speciation in Atlantic eels (*Anguilla anguilla* and *A. rostrata*). Mol. Ecol. 23, 4785–4798. 10.1111/mec.12896 25155907

[B19] Kirk R. S. (2003). The impact of *Anguillicola crassus* on European eels. Fish. Manag. Ecol. 10, 385–394. 10.1111/j.1365-2400.2003.00355.x

[B20] Kleckner R. C. (1980a). Swim bladder volume maintenance related to migratory depth in silver phase *Anguilla rostrata*. Science 208, 1481–1482. 10.1126/science.7384792 7384792

[B21] Kleckner R. C. (1980b). Swimbladder wall guanine enhancement related to migratory depth in silver phase *Anguilla rostrata*. Comp. Biochem. Physiol. 65A, 351–354. 10.1016/0300-9629(80)90041-9

[B22] Kobayashi H. Pelster B. Scheid P. (1990). CO_2_ back-diffusion in the rete aids O_2_ secretion in the swimbladder of the eel. Respir. Physiol. 79, 231–242. 10.1016/0034-5687(90)90129-M 2113304

[B23] Lefebvre F. Fazio G. Mounaix B. Crivelli A. J. (2013). Is the continental life of the European eel *Anguilla anguilla* affected by the parasitic invader *Anguillicoloides crassus*? Proc. R. Soc. B Biol. Sci. 280:2012.2916. 10.1098/rspb.2012.2916 23325776 PMC3574337

[B24] Lefebvre F. Fazio G. Palstra A. P. Székely C. Crivelli A. J. (2011). An evaluation of indices of gross pathology associated with the nematode *Anguillicoloides crassus* in eels. J. Fish Dis. 34, 31–45. 10.1111/j.1365-2761.2010.01207.x 21118268

[B25] Li H. Handsaker B. Wysoker A. Fennell T. Ruan J. Homer N.. (2009). The sequence alignment/map format and SAMtools. Bioinformatics 25, 2078–2079. 10.1093/bioinformatics/btp352 19505943 PMC2723002

[B26] Lushchak V. I. Semchyshyn H. M. (2012). Oxidative Stress - Molecular Mechanisms and Biological Effects. Rijeka: InTech 10.5772/2333

[B27] Molnár K. Baska F. Csaba G. Glávitis R. Székely C. (1993). Pathological and histopathological studies of the swimbladder of eels *Anguilla anguilla* infected by *Anguillicola crassus* (Nematoda, Dracunculoidea). Dis. Aquat. Org. 15, 41–50. 10.3354/dao015041

[B28] Molnár K. Szakolczai J. Vetési F. (1995). Histological changes in the swimbladder wall of eels due to abnormal location of adults and second stage larvae of *Anguillicola crassus*. Acta Vet. Hung. 43, 125–137. 7625284

[B29] Morris S. M. Albright J. T. (1981). Superoxide dismutase, catalase, and glutathione peroxidase in the swim bladder of the physoclistous fish, *Opsanus tau* L. Cell Tiss. Res. 220, 739–752. 10.1007/BF00210458 7296650

[B30] Morris S. M. Albright J. T. (1984). Catalase, glutathione peroxidase, and superoxide dismutase in the rete mirabile and gas gland epithelium of six species of marine fishes. J. Exp. Zool. 232, 29–39. 10.1002/jez.1402320105 6502092

[B31] Nimeth K. Zwerger P. Würtz J. Salvenmoser W. Pelster B. (2000). Infection of the glass-eel swimbladder with the nematode *Anguillicola crassus*. Parasitology 121, 75–83. 10.1017/S003118209900606X 11085227

[B32] Pelster B. (1995a). Mechanisms of acid release in isolated gas gland cells of the European eel *Anguilla anguilla*. Am. J. Physiol. 269, R793–R799. 7485595 10.1152/ajpregu.1995.269.4.R793

[B33] Pelster B. (1995b). Metabolism of the swimbladder tissue. Biochem. Mol. Biol. Fish. 4, 101–118. 10.1016/S1873-0140(06)80008-1

[B34] Pelster B. (1997). Buoyancy at depth, in Deep-Sea Fish, eds Randall D. Farrell A. P. (San Diego, CA: Academic Press), 195–237.

[B35] Pelster B. (2001). The generation of hyperbaric oxygen tensions in fish. News Physiol. Sci. 16, 287–291. 11719607 10.1152/physiologyonline.2001.16.6.287

[B36] Pelster B. (2009). Buoyancy control in aquatic vertebrates, in Cardio-Respiratory Control in Vertebrates, eds Glass M. L. Wood S. C. (Berlin/Heidelberg: Springer Verlag), 65–98.

[B37] Pelster B. (2013). The swimbladder, in Eel Physiology, eds Trischitta F. Takei Y. Sebert P. (Enfield: CRC Press), 44–67.

[B38] Pelster B. (2015). Swimbladder function and the spawning migration of the European eel *Anguilla anguilla*. Front. Physiol. 5:486. 10.3389/fphys.2014.00486 25646080 PMC4297919

[B39] Pelster B. Hicks J. Driedzic W. R. (1994). Contribution of the pentose phosphate shunt to the formation of CO_2_ in swimbladder tissue of the eel. J. Exp. Biol. 197, 119–128. 9317460 10.1242/jeb.197.1.119

[B40] Pelster B. Randall D. J. (1998). The physiology of the root effect, in Fish Respiration, eds Perry S. F. Tufts B. L. (San Diego, CA: Academic Press), 113–139.

[B41] Pelster B. Scheid P. (1993). Glucose metabolism of the swimbladder tissue of the European eel *Anguilla anguilla*. J. Exp. Biol. 185, 169–178.

[B42] Pelster B. Weber R. E. (1991). The physiology of the root effect. Adv. Comp. Environ. Physiol. 8, 51–77. 10.1007/978-3-642-75900-0_2

[B43] Righton D. Aarestrup K. Jellyman D. Sébert P. van den Thillart G. Tsukamoto K. (2012). The *Anguilla* spp. migration problem: 40 million years of evolution and two millennia of speculation. J. Fish Biol. 81, 365–386. 10.1111/j.1095-8649.2012.03373.x 22803715

[B44] Schneebauer G. Hanel R. Pelster B. (2016). *Anguillicola crassus* impairs the silvering-related enhancements of the ROS defense capacity in swimbladder tissue of the European eel (*Anguilla anguilla*). J. Comp. Physiol. B. 10.1007/s00360-016-0994-0. [Epub ahead of print]. 27146148 PMC5009179

[B45] Sebert P. Vettier A. Amerand A. Moisan C. (2009). High pressure resistance and adaptation of European eels, in Spawning Migration of the European Eel, eds van den Thillart G. Dufour S. Rankin J. C. (New York, NY: Springer Verlag), 99–127.

[B46] Steen J. B. (1963). The physiology of the swimbladder in the eel *Anguilla vulgaris*. III. The mechanism of gas secretion. Acta Physiol. Scand. 59, 221–241. 10.1111/j.1748-1716.1963.tb02738.x 14078660

[B47] Supuran C. T. (2008). Carbonic anhydrases: novel therapeutic applications for inhibitors and activators. Nat. Rev. Drug Discov. 7, 168–181. 10.1038/nrd2467 18167490

[B48] Trapnell C. Pachter L. Salzberg S. L. (2009). TopHat: discovering splice junctions with RNA-Seq. Bioinformatics 25, 1105–1111. 10.1093/bioinformatics/btp120 19289445 PMC2672628

[B49] Walsh P. J. Milligan C. L. (1993). Roles of buffering capacity and pentose phosphate pathway activity in the gas gland of the gulf toadfish *Opsanus beta*. J. Exp. Biol. 176, 311–316.

[B50] Wang H. Gong Z. (1999). Characterization of two zebrafish cDNA clones encoding egg envelope proteins ZP2 and ZP3. Biochim. Biophys. Acta 1446, 156–160. 10.1016/S0167-4781(99)00066-4 10395930

[B51] Würtz J. Salvenmoser W. Pelster B. (1999). Localization of carbonic anhydrase in swimbladder tissue of European eel (*Anguilla anguilla*) and perch (*Perca fluviatilis*). Acta Physiol. Scand. 165, 219–224. 10.1046/j.1365-201x.1999.00501.x 10090334

[B52] Würtz J. Taraschewski H. (2000). Histopathological changes in the swimbladder wall of the European eel *Anguilla anguilla* due to infections with *Anguillicola crassus*. Dis. Aquat. Org. 39, 121–134. 10.3354/dao039121 10715817

[B53] Würtz J. Taraschewski H. Pelster B. (1996). Changes in gas composition in the swimbladder of the European eel (*Anguilla anguilla*) infected with *Anguillicola crassus* (Nematoda). Parasitology 112, 233–238. 10.1017/S003118200008481X 8851864

[B54] Wysujack K. Westerberg H. Aarestrup K. Trautner J. Kurwie T. Nagel F. (2015). The migration behaviour of European silver eels (*Anguilla anguilla*) released in open ocean conditions. Mar. Freshwater Res. 66, 145–157. 10.1071/MF14023

[B55] Yamada Y. Zhang H. Okamura A. Tanaka S. Horie N. Mikawa N. (2001). Morphological and histological changes in the swim bladder during maturation of the Japanese eel. J. Fish. Biol. 58, 804–814. 10.1111/j.1095-8649.2001.tb00532.x

