# Supplementary material for: Anguillicola crassus Infection Significantly Affects the Silvering Related Modifications in Steady State mRNA Levels in Gas Gland Tissue of the European Eel
Source: Front Physiol. 2016 May 23;7:175. doi: 10.3389/fphys.2016.00175 (PMC4876612; doi:10.3389/fphys.2016.00175)
Supplement: Supplementary file 1 [file Table1.pdf]

Supplementary Table 1

Differentially transcribed genes based on GO terms response to ion exchange in uninfected and in infected swimbladder silver eel swimbladder tissue as compared to uninfected yellow eel swimbladder tissue (*P* < 0.01).

			Uninfected silver	Infected silver
**Gene**	**Name**	**Description**	**fold change**	**fold change**
g10634	nhrf3	na(+) h(+) exchange regulatory cofactor	110.52	
g881	mypr	myelin proteolipid protein	15.25	
g38167	p3	p3 protein	7.85	
g24053	gima4	gtpase imap family member 4	6.81	
g17810	atng	sodium potassium-transporting atpase subunit gamma	6.22	
g2281	kcnb1	potassium volt.-gated ch. subf. b member 1	6.10	
g27889	fyn	tyrosine-protein kinase fyn	5.69	
g14408	p3	p3 protein	4.12	
g11852	hfe	hereditary hemochromatosis protein	0.55	
g12358	so2a1	org. anion transp. family member 2a1	0.27	
g649	cftr	cystic fibrosis transm. conduct. reg.	0.18	
g18164	s28a3	solute carrier family 28 member 3	0.16	
g24196	gbrb2	gamma-aminob. acid rec. subunit beta-2	0.08	
g10461	s39a8	zinc transporter zip8	0.07	
g26738	hfe	hereditary hemochromatosis protein	0.04	
g16142	ticn1	testican-1	0.03	
g9336	cac1e	voltage-dep. r-type calcium ch. sub. alpha-1e	0.00	
g12086	l3bpb	galectin-3-binding protein b	57.66	35.14
g25543	gima7	gtpase imap family member 7	52.70	5.32
g14635	gima7	gtpase imap family member 7	20.87	3.79
g20433	scar5	scavenger receptor class a member 5	7.28	4.09
g21824	addb	beta-adducin	0.14	0.23
g11712	nduv1	nadh dehyd. flavoprot. mitochondrial	0.14	0.13
g23227	sl9a1	sodium hydrogen exchanger 1	0.13	0.17
g17474	grik1	glutamate ionotropic kainate 1	0.12	0.06
g21073	grik1	glutamate ionotropic kainate 1	0.08	0.03
g37728	b3a2	anion exchange protein 2 short	0.04	0.17
g30027	s6a12	sod.- and chloride-depend. betaine transp.	0.02	0.03
g11255	wisp3	wnt1-inducible-signal. pathw. protein 3		Inf
g18418	sc5a1	sodium glucose cotransporter 1		44.42
g40336	hpt	haptoglobin contains:		37.95
g3715	lpar6	lysophosphatidic acid receptor 6		21.90
g3803	grik2	glutamate ionotropic kainate 2		13.29

g18935	p2ry4	p2y purinoceptor 4 short=p2y4	10.76
g11761	s13a3	solute carrier family 13 member 3	9.90
g4046	stea4	metalloreductase steap4	9.79
g7770	mt12b	monocarboxylate transporter 12-b	8.54
g23306	gima4	gtpase imap family member 4	6.35
g20867	s6a15	orphan sod.- and chl.-dep. neurotransm. transp. ntt73	5.80
g9003	vatg1	v-type proton atpase subunit g 1	4.27
g22693	cor1a	coronin-1a	4.18
g2350	eaa2	excitatory amino acid transporter 2	4.03
g1407	hck	tyrosine-protein kinase hck	3.90
g21858	cor1a	coronin-1a	3.83
g21400	kcma1	calcium-activated pot. ch. subunit alpha-1	0.21
g7353	so2a1	organic anion transporter family member 2a1	0.20
g7841	at1b2	sod. pot.-transp. atpase subunit beta-2	0.14
g28265	actn3	alpha-actinin-3	0.11
g17469	accn1	amiloride-sensitive cation channel neuronal	0.02
g22000	actn3	alpha-actinin-3	0.00
g406	so1c1	organic anion transp. fam. memb. 1c1	0.00
g42674	jph2	junctophilin-2	0.00
