# Supplementary material for: Anguillicola crassus Infection Significantly Affects the Silvering Related Modifications in Steady State mRNA Levels in Gas Gland Tissue of the European Eel
Source: Front Physiol. 2016 May 23;7:175. doi: 10.3389/fphys.2016.00175 (PMC4876612; doi:10.3389/fphys.2016.00175)
Supplement: Supplementary file 2 [file Table2.pdf]

Supplementary Table 2

Differentially transcribed genes based on GO terms related to immune response in uninfected and in infected swimbladder silver eel swimbladder tissue as compared to uninfected yellow eel swimbladder tissue (*P* < 0.01).

			Uninfected silver	Infected silver
**Gene**	**Name**	**Description**	**fold change**	**fold change**
g21158	cytc	cystatin-c	Inf	
g40079		immunoglobulin light chain	464.69	
g31085	gbp5	guanylate-binding protein 5	95.19	
g31792	lv302	ig lambda chain v-iii region loi	89.92	
g1076	cats	cathepsin s flags: precursor	28.09	
g26738	nptx1	neuronal pentraxin-1	19.97	
g23965	lac6	ig lambda-6 chain c region	18.12	
g34285	lv302	ig lambda chain v-iii region loi	13.16	
g19117	pa2gx	group 10 secretory phospholipase a2	12.94	
g35655	cxl10	c-x-c motif chemokine 10	12.82	
g28118	scub1	signal cub and egf-like domain-cont protein 1	11.63	
g36679	galt8	prob polypepti n-acetylgalactosaminyltransferase 8	11.26	
g14663	cp1b1	cytochrome p450 1b1	11.19	
g14172	cadm2	cell adhesion molecule 2	10.89	
g31640	ha1k	h-2 class i histocomp k-k alpha chain	10.86	
g278	tnfa	tumor necrosis factor	9.70	
g24963	tcb	t-cell receptor beta chain t17t-22	9.68	
g15375	ddn1	duodenase-1	9.24	
g25978	iigp5	interferon-inducible gtpase 5	9.09	
g16242	cfab	complement factor b	8.11	
g24694	mmp17	matrix metalloproteinase-17	8.09	
g8995	scub2	signal cub and egf-like domain-containing protein 2	7.87	
g33491	tcb2	t-cell receptor beta-2 chain c region	6.84	
g24053	gima4	gtpase imap family member 4	6.81	
g37466	hmr1	major histocomp. compl. class i-related gene protein	6.73	
g41695	hmr1	major histocomp. compl.class i-related gene protein	6.14	
g13207	c1qc	complement c1q subcomponent subunit c	6.10	
g13774	ap1s2	ap-1 complex subunit sigma-2	5.88	
g11997	in35	interferon-induced 35 kda protein	5.76	
g27889	fyn	tyrosine-protein kinase fyn	5.69	
g41499	tpsnr	tapasin-related protein	5.64	
g40645	galt8	probable polypeptide n-acetylgalactosaminyltransf. 8	5.37	
g19746	gbp4	guanylate-binding protein 4	4.61	

g10119	mbl2	mannose-binding protein c	4.60	
g20076	cd3g	t-cell surface glycoprotein cd3 gamma chain	4.27	
g11852	hfe	hereditary hemochromatosis protein	0.55	
g11202	crld2	cysteine-rich secret. protein lccl domain-cont. 2 flags	0.17	
g27549	ita2	integrin alpha-2	0.07	
g26738	hfe	hereditary hemochromatosis protein	0.04	
g31917	ita10	integrin alpha-10	0.04	
g16142	ticn1	testican-1	0.03	
g15394	inhbb	inhibin beta b chain	0.02	
g22923	cfah	complement factor h	Inf	Inf
g49	sem3f	semaphorin-3f	Inf	Inf
g30915	mx	interferon-induced gtp-binding protein mx	94.94	79.38
g19950	il17b	interleukin-17b short=il-17b	81.28	24.10
g39956	mx	interferon-induced gtp-binding protein mx	70.50	91.94
g12085	l3bpb	galectin-3-binding protein b	62.85	36.90
g12086	l3bpb	galectin-3-binding protein b	57.66	35.14
g4496	ddx58	probable atp-dependent rna helicase ddx58	54.76	28.23
g25543	gima7	gtpase imap family member 7	52.70	5.32
g45177	l3bpb	galectin-3-binding protein b	52.41	38.33
g43530	mx	interferon-induced gtp-binding protein mx	49.45	50.50
g17524	rsad2	radical s-adenosyl methionine domain-cont protein 2	48.39	23.50
g15237	par12	poly polymerase 12	45.11	31.02
g18110	par11	poly polymerase 11	44.06	34.90
g14949	co3	complement c3 contains:	43.98	16.47
g2962	dhx58	probable atp-dependent rna helicase dhx58	35.96	25.42
g9510	herc3	probable e3 ubiquitin-protein ligase herc3	35.87	30.77
g17733	fhr2	complement factor h-related protein 2	33.65	6.52
g8265	frim	middle subunit ferritin	30.39	6.20
g26753	cxl11	c-x-c motif chemokine 11	30.20	5.24
g11604	ccl19	c-c motif chemokine 19	29.56	12.07
g14950	co3	complement c3 contains:	23.26	109.15
g39396	ifi44	interferon-induced protein 44	22.93	11.07
g26685	mx	interferon-induced gtp-binding protein mx	22.53	22.35
g13181	ifi44	interferon-induced protein 44	22.09	8.90
g12107	grn	granulins	20.87	5.16
g14635	gima7	gtpase imap family member 7	20.87	3.79
g36894	lysc2	lysozyme c ii	20.18	9.05
g1090	ileu	leukocyte elastase inhibitor	19.71	4.25
g34980	lysc3	lysozyme c-3	19.13	10.12
g23857	tri16	tripartite motif-containing protein 16	17.85	11.83
g40174	mx	interferon-induced gtp-binding protein mx	17.77	17.05
g11629	ddx58	probable atp-dependent rna helicase ddx58	17.46	12.56
g28341		novel prot verteb interferon-induced protein 44	16.88	4.67
g12275	irf7	interferon regulatory factor 7	16.24	16.11

g26148	if44l	interferon-induced protein 44-like	15.29	11.77
g7750	tsp4b	thrombospondin-4-b	15.23	27.87
g533	tlr13	toll-like receptor 13 flags: precursor	13.99	5.53
g6958	stat1	signal transducer and activator of transcription 1	13.70	11.91
g2101	thms1	protein themis	13.60	5.72
g4842	oxla	l-amino-acid oxidase	13.48	13.22
g1450	cd22	b-cell receptor cd22	12.48	11.69
g38311	tri29	tripartite motif-containing protein 29	12.10	6.51
g43250	tri16	tripartite motif-containing protein 16	11.64	4.86
g12933	scub1	signal cub and egf-like dom-contain protein 1	11.61	28.16
g38983	par12	poly polymerase 12	11.58	8.08
g36915	tri29	tripartite motif-containing protein 29	11.29	6.14
g38218	ddx58	probable atp-dependent rna helicase ddx58	11.29	8.35
g11916	tcc4	t-cell receptor gamma chain c region 5 10-13	10.92	4.57
g11223	lyve1	lymphatic vessel endoth hyaluronic acid receptor 1	10.63	12.21
g44763	tri29	tripartite motif-containing protein 29	10.60	6.42
g17105	h2az	histone h2a	10.45	4.76
g24958	tri25	e3 ubiquitin isg15 ligase trim25	10.35	6.09
g27564	nmi	n-myc-interactor short=nmi	9.27	5.79
g23858	tri25	e3 ubiquitin isg15 ligase trim25	9.20	6.27
g18710	e2ak2	interf- double-strand rna-activated prot kinase	9.18	5.30
g1848	ccl25	c-c motif chemokine 25	8.85	8.35
g26078	cxcl2	c-x-c motif chemokine 2	8.67	7.37
g36895	mx2	interferon-induced gtp-binding protein mx2	8.57	5.22
g28207	gima7	gtpase imap family member 7	7.81	7.22
g10354	bcl6	b-cell lymphoma 6 protein homolog	7.37	7.50
g20433	scar5	scavenger receptor class a member 5	7.28	4.09
g26875	lysc	lysozyme c	7.13	6.77
g30678	par12	poly polymerase 12	6.24	10.49
g20440	irf8	interferon regulatory factor 8	6.17	3.81
g24959	tri29	tripartite motif-containing protein 29	5.89	5.02
g13206	fbx40	f-box only protein 40	5.78	3.78
g14226	a33	zinc-binding protein a33	5.51	4.82
g18684	casp1	caspase-1	5.40	4.58
g41989	cflar	casp8 and fadd-like apoptosis regulator	5.31	5.28
g4949	lyg	lysozyme g	5.08	5.78
g22287	par12	poly polymerase 12	4.19	4.67
g22618	chia	acidic mammalian chitinase	3.84	13.40
g16986	h13	histone	0.25	0.09
g3963	ncam2	neural cell adhesion molecule 2	0.20	0.26
g17014	nal12	lrr and pyd domains-containing protein 12	0.14	0.10
g12409	fos	proto-oncogene c-fos	0.08	0.07
g28238	pvrl3	poliovirus receptor-related protein 3-like	0.08	0.09
g3322	fos	proto-oncogene c-fos	0.08	0.21

g11642	cof2	cofilin-2	0.06	0.05
g11898	fosb	protein fosb	0.05	0.08
g32679	alsi	alpha-1-antitrypsin-like protein cm55-si	0.00	0.05
g513	sem3c	semaphorin-3c		Inf
g22125	twhh	tiggy-winkle hedgehog protein		Inf
g35363	muc5a	mucin-5ac		Inf
g37402	muc5b	mucin-5b		Inf
g6564	prg4	proteoglycan 4		73.72
g2358	noxo1	nadph oxidase organizer 1		49.96
g34568	muc5a	mucin-5ac		38.60
g40336	hpt	haptoglobin contains:		37.95
g5825	col10	collectin-10		29.07
g6637	cy24b	cytochrome b-245 heavy chain		27.50
g3715	lpar6	lysophosphatidic acid receptor 6		21.90
g12811	co3	complement c3 contains:		20.61
g23617	chia	acidic mammalian chitinase		14.90
g38450	bcl6	b-cell lymphoma 6 protein		11.23
g30308	dclk2	serine threonine-protein kinase dclk2		10.25
g20378	trhde	thyrotropin-releasing hormone-degrading ectoenzyme		9.75
g4763		interferon-induced protein 44-like		8.73
g2443	rgs4	regulator of g-protein signaling 4		8.29
g3149	ednrb	endothelin b receptor		8.02
g22925	dclk2	serine threonine-protein kinase dclk2		7.81
g838	ptx3	pentraxin-related protein ptx3		7.16
g13857	s7a14	probable cationic amino acid transporter		7.11
g12175	enob	beta-enolase		6.96
g36054	s1pr4	sphingosine 1-phosphate receptor 4		6.79
g25084	pnph	purine nucleoside phosphorylase		6.51
g23306	gima4	gtpase imap family member 4		6.35
g24330	dclk2	serine threonine-protein kinase dclk2		6.32
g5914	dmbt1	deleted in malignant brain tumors 1 protein		6.08
g7735	cy24b	cytochrome b-245 heavy chain		5.67
g7816	hmox	heme oxygenase		5.53
g29481	mk11	mitogen-activated protein kinase 11		5.51
g10869	hemo	hemopexin flags: precursor		5.46
g386	slap1	src-like-adapter		5.34
g21455	pnph	purine nucleoside phosphorylase		5.03
g7584	cxcr1	c-x-c chemokine receptor type 1		4.81
g15498	lox5	arachidonate 5-lipoxygenase		4.73
g20618	il6rb	interleukin-6 receptor subunit beta		4.59
g25978	iigp5	interferon-inducible gtpase 5		4.51
g9598	pxdn	peroxidasin homolog flags: precursor		4.47
g15581	tecta	alpha-tectorin flags: precursor		4.46
g15158	apj	apelin receptor		4.38

g44679	co5	complement c5	4.37
g24625	clm3	cmrf35-like molecule 3	4.34
g23573	gbp6	guanylate-binding protein 6	4.32
g7116	c5ar	c5a anaphylatoxin chemotactic receptor	4.32
g19805	rhog	rho-related gtp-binding protein flags: precursor	4.27
g22693	cor1a	coronin-1a	4.18
g22496	fyb	fyn-binding protein	4.16
g12898	ptprh	receptor-type tyrosine-protein phosphatase h	4.15
g18927	urom	uromodulin	4.13
g10479	ncf1	neutrophil cytosol factor 1	4.04
g26433	ccr4	c-c chemokine receptor type 4	4.00
g32070	hmr1	major histocompatibility complex class i-related gene protein	3.99
g14118	tri25	e3 ubiquitin isg15 ligase trim25	3.97
g17465	cy24a	cytochrome b-245 light chain	3.94
g8065	clc4e	c-type lectin domain family 4 member e	3.90
g1407	hck	tyrosine-protein kinase hck	3.90
g21858	cor1a	coronin-1a	3.83
g22559	hxk2	hexokinase-2	3.83
g10077	c1r	complement c1r subcomponent	3.82
g31471	cats	cathepsin s flags: precursor	3.81
g1493	rgs8	regulator of g-protein signaling 8	3.80
g30240	plsl	plastin-2	3.73
g10742	cdo1	cysteine dioxygenase type 1	3.64
g30264	plsl	plastin-2	3.56
g20847	myo1f	myosin-if	3.53
g27501	cats	cathepsin s flags: precursor	3.49
g3631	nk3r	neuromedin-k receptor	0.25
g27556	if4h	eukaryotic translation initiation factor 4h	0.22
g9834	cadm1	cell adhesion molecule 1	0.22
g16879	tmps6	transmembrane protease serine 6	0.18
g8735	ssr1	somatostatin receptor type 1	0.15
g2030	pa24c	cytosolic phospholipase a2 gamma	0.15
g28238	pvrl3	poliovirus receptor-related protein 3-like flags	0.09
g26554	gima7	gtpase imap family member 7	0.09
g1679	actc	alpha cardiac	0.00
