# Supplementary material for: Anguillicola crassus Infection Significantly Affects the Silvering Related Modifications in Steady State mRNA Levels in Gas Gland Tissue of the European Eel
Source: Front Physiol. 2016 May 23;7:175. doi: 10.3389/fphys.2016.00175 (PMC4876612; doi:10.3389/fphys.2016.00175)
Supplement: Supplementary file 3 [file Table3.pdf]

Supplementary Table 3

Differentially transcribed genes based on GO terms related to sexual maturation in uninfected and in infected swimbladder silver eel swimbladder tissue as compared to uninfected yellow eel swimbladder tissue (*P* < 0.01).

			Uninfected silver	Infected silver
**Gene**	**Name**	**Description**	**fold change**	**fold change**
g18804	ccna1	cyclin-a1	Inf	
g1804	ima1	importin subunit alpha-1	Inf	
g19899	k1c18	type i cytoskeletal 18	Inf	
g23807	mmp13	collagenase 3	Inf	
g1675	sox3	transcription factor sox-3	Inf	
g15786	zp1	zona pellucida sperm-binding protein 1	Inf	
g13702	zp3	zona pellucida sperm-binding protein 3	229.78	
g45297	zp2	zona pellucida sperm-binding protein 2	226.92	
g36576	zp3	zona pellucida sperm-binding protein 3	214.73	
g16004	zp3	zona pellucida sperm-binding protein 3	178.67	
g10976	cd9	cd9 antigen ame: cd_antigen=cd9	25.68	
g18169	k1c13	type i cytoskeletal 13	15.41	
g1568	b4	protein b4	13.18	
g14663	cp1b1	cytochrome p450 1b1	11.19	
g11796	k1c13	type i cytoskeletal 13	11.16	
g24694	mmp17	matrix metalloproteinase-17	8.09	
g13550	dus5	dual specificity protein phosphatase 5	7.23	
g28210	mycbp	c-myc-binding protein	6.80	
g27889	fyn	tyrosine-protein kinase fyn	5.69	
g23663	fhdc1	fh2 domain-containing protein 1	4.98	
g36354	par14	poly polymerase 14	4.77	
g6349	tshr	thyrotropin receptor	0.25	
g27891	rdh8	retinol dehydrogenase 8	0.20	
g9306	dnjb4	homolog subfamily b member 4	0.20	
g649	cftr	cystic fibrosis transmembrane conductance regulator	0.18	
g13602	angp1	angiopoietin-1	0.17	
g12711	mmp9	matrix metalloproteinase-9	0.08	
g22791	herc4	probable e3 ubiquitin-protein ligase herc4	58.30	39.60
g15237	par12	poly polymerase 12	45.11	31.02
g18110	par11	poly polymerase 11	44.06	34.90

g9510	herc3	probable e3 ubiquitin-protein ligase herc3	35.87	30.77
g2311	ccnb1	g2 mitotic-specific cyclin-b1	22.34	13.87
g1090	ileu	leukocyte elastase inhibitor	19.71	4.25
g7571	ccna2	cyclin-a2	17.98	13.72
g23857	tri16	tripartite motif-containing protein 16	17.85	11.83
g977	al1a3	aldehyde dehydrogenase family 1 member a3	17.28	10.83
g6324	esr2	estrogen receptor beta	15.63	6.55
g7750	tsp4b	thrombospondin-4-b	15.23	27.87
g20773	m10b1	helicase mov-10-	14.90	16.49
g6958	stat1	signal transducer and activator of transcription 1	13.70	11.91
g38311	tri29	tripartite motif-containing protein 29	12.10	6.51
g38983	par12	poly polymerase 12	11.58	8.08
g36915	tri29	tripartite motif-containing protein 29	11.29	6.42
g44763	tri29	tripartite motif-containing protein 29	10.60	6.14
g24958	tri25	e3 ubiquitin isg15 ligase trim25	10.35	6.27
g40263	m10b2	helicase mov-10-	9.63	15.39
g5447	socs1	suppressor of cytokine signaling 1	9.60	5.04
g23858	tri25	e3 ubiquitin isg15 ligase trim25	9.20	6.09
g19804	a33	zinc-binding protein a33	8.18	17.76
g9146	stat1	signal transducer and activator of transcription 1	7.63	8.82
g10354	bcl6	b-cell lymphoma 6 protein homolog	7.37	7.50
g24959	tri29	tripartite motif-containing protein 29	5.89	5.02
g14226	a33	zinc-binding protein a33	5.51	4.82
g41989	cflar	casp8 and fadd-like apoptosis regulator	5.31	5.28
g16888	tdrd7	tudor domain-containing protein 7	5.01	5.54
g11406	ldlr2	low-density lipoprotein receptor 2	4.75	5.24
g16986	h13	histone	0.25	0.09
g22978	socs2	suppressor of cytokine signaling 2	0.17	0.27
g17014	nal12	lrr and pyd domains-containing protein 12	0.14	0.10
g21896	ank1	ankyrin-1	0.12	0.11
g12409	fos	proto-oncogene c-fos	0.08	0.21
g3322	fos	proto-oncogene c-fos	0.08	0.07
g11898	fosb	protein fosb	0.05	0.08
g13055	tmpsd	transmembrane protease serine 13	0.04	0.16
g30027	s6a12	sodium- and chloride-dependent betaine transporter	0.02	0.03
g32210	hp55	hibernation-specific plasma protein hp-55	0.01	0.04
g19787	hp55	hibernation-specific plasma protein hp-55	0.00	0.02
g32679	alsi	alpha-1-antitrypsin-like protein cm55-si	0.00	0.05
g22125	twhh	tiggy-winkle hedgehog protein		Inf
g11255	wisp3	wnt1-inducible-signaling pathway protein 3		Inf
g9322	dus27	inactive dual specificity phosphatase 27		24.06
g13985	tdrd1	tudor domain-containing protein 1		17.07
g12188	aurkb	serine threonine-protein kinase 12		8.72
g27065	avt	-vasotocin receptor		7.90

g15582	gp112	probable g-protein coupled receptor 112	6.44
g5914	dmbt1	deleted in malignant brain tumors 1 protein	6.08
g8653	fr1l4	fer-1-like protein 4	5.74
g7816	hmox	heme oxygenase	5.53
g2441	rgs8	regulator of g-protein signaling 8	5.18
g21556	st2b1	sulfotransferase family cytosolic 2b member 1	4.99
g23866	sox8	transcription factor sox-8	4.68
g15581	tecta	alpha-tectorin flags: precursor	4.46
g19805	rhog	rho-related gtp-binding protein	4.27
g14118	tri25	e3 ubiquitin isg15 ligase trim25	3.97
g5037	pkh4b	pleckstrin homology domain-containing family g member 4b	3.97
g3631	nk3r	neuromedin-k receptor	0.25
g7089	itr	isotocin receptor	0.25
g21703	s22ag	solute carrier family 22 member 16	0.22
g7353	so2a1	solute carrier organic anion transporter family member 2a1	0.20
g9834	cadm1	cell adhesion molecule 1	0.22
g25479	g3st1	galactosylceramide sulfotransferase	0.19
g24544	nr0b2	nuclear receptor subfamily 0 group b member 2	0.17
g3034	socs2	suppressor of cytokine signaling 2	0.13
g3911	anr45	ankyrin repeat domain-containing protein 45	0.11
g14472	whrn	whirlin	0.06
g20026	dupd1	dual specificity phosphatase dupd1	0.05
